# Effects on muscle performance of NSAID treatment with Piroxicam versus placebo in geriatric patients with acute infection-induced inflammation. a double blind randomized controlled trial

**DOI:** 10.1186/1471-2474-12-292

**Published:** 2011-12-30

**Authors:** Ingo Beyer, Ivan Bautmans, Rose Njemini, Christian Demanet, Pierre Bergmann, Tony Mets

**Affiliations:** 1 Department of Geriatrics, Universitair Ziekenhuis Brussel, Laarbeeklaan 101, B-1090 Brussels, Belgium; 2 Frailty in Aging Research Group (FRIA) & Gerontology department, Vrije Universiteit Brussel (VUB), Laarbeeklaan 103, Brussels B-1090, Belgium; 3 Department of HLA & Molecular Hematology, Universitair Ziekenhuis Brussel, Laarbeeklaan 101, Brussels, Belgium; 4 Department of Nuclear Medicine, CHU Brugmann, Université Libre de Bruxelles (ULB), 4 Place Van Gehuchten, Brussels B-1020, Belgium

**Keywords:** muscle performance, piroxicam, NSAID, cytokines, heat shock protein

## Abstract

**Background:**

Inflammation is the main cause of disease-associated muscle wasting. In a previous single blind study we have demonstrated improved recovery of muscle endurance following celecoxib treatment in hospitalized geriatric patients with acute infection. Here we further evaluate NSAID treatment with piroxicam in a double blind RCT and investigate the role of cytokines and heat shock proteins (Hsp) with respect to muscle performance. We hypothesized that NSAID treatment would preserve muscle performance better than antibiotic treatment alone, by reducing infection-associated inflammation and by increasing expression of cytoprotective Hsp.

**Methods:**

Consecutive admissions to the geriatric ward were screened. 30 Caucasian patients, median age 84.5 years, with acute infection-induced inflammation and serum levels of CRP > 10 mg/L were included and randomized to active treatment with 10 mg piroxicam daily or placebo. Assessment comprised general clinical and biochemical parameters, 25 cytokines in serum, intra-and extracellular Hsp27 and Hsp70, Elderly Mobility Scale (EMS) scores, grip strength (GS), fatigue resistance (FR) and lean body mass (LBM). Patients were evaluated until discharge with a maximum of 3 weeks after treatment allocation.

**Results:**

EMS scores, FR and grip work (GW), a measure taking into account GS and FR, significantly improved with piroxicam, but not with placebo. Early decreases in IL-6 serum levels with piroxicam correlated with better muscle performance at week 2. Basal expression of Hsp27 in monocytes without heat challenge (WHC) was positively correlated with FR at baseline and significantly increased by treatment with piroxicam compared to placebo. Profound modifications in the relationships between cytokines or Hsp and changes in muscle parameters were observed in the piroxicam group.

**Conclusions:**

Piroxicam improves clinically relevant measures of muscle performance and mobility in geriatric patients hospitalized with acute infection-induced inflammation. Underlying mechanisms may include modifications in the cytokine network and increases in monocytic expression of cytoprotective Hsp27.

**Trial registration number:**

ISRCTN: ISRCTN96340690

## Background

Sarcopenia, the age-related loss of muscle mass and performance, is a major component of frailty [1]. It is aggravated by acute disease-induced muscle wasting, precipitating functional decline during and after hospitalization. Inflammation with its accompanying cytokine production is seen as a predominant cause of muscle wasting due to chronic or acute disease [2]. Previously, we have shown that acute inflammation hinders the recovery of muscle performance in geriatric hospitalized patients [3] and in elderly elective surgery patients [4]. Besides treatment of the underlying acute disease, interventions able to reduce inflammation-related adverse outcomes may be warranted. We have previously demonstrated a better recovery of muscle endurance following treatment with celecoxib, a cyclooxygenase(COX)-2 selective drug, in hospitalized geriatric patients with acute infection [5]. However, in that study only a few inflammatory mediators were studied. Non selective non steroidal anti-inflammatory drugs (NSAID), including piroxicam, have been shown to inhibit lipopolysaccharide(LPS)-induced expression of cytokines involved in macrophage activation and in the acute phase response, while activating stress genes and increasing the expression of heat shock proteins (Hsp) [6]. Cell-protective mechanisms, such as increased production of Hsp, are blunted in muscle tissues of older patients [7]. In the present placebo-controlled randomized controlled trial (RCT) we investigated the effect of piroxicam on the evolution of muscle performance and mobility in hospitalized geriatric patients with acute infection. A large set of circulating cytokines and chemokines as well as intra-and extra-cellular Hsp were measured. We hypothesized that NSAID treatment would preserve muscle performance by reducing infection-induced inflammation faster than antibiotics alone, and possibly by increasing expression of Hsp. Our results show improvements in muscle performance and basic mobility with piroxicam, but not with placebo.

## Methods

### Participants

Patients aged ≥ 70 years, consecutively admitted to an acute geriatric ward of a general teaching hospital for acute infection (documented by C-reactive protein (CRP) serum level > 10 mg/L and/or fibrinogen > 400 mg/dL), were eligible. Patients presenting inflammation of non-infectious origin, using corticosteroids or NSAID within the past seven days, or showing contra-indication to treatment were excluded, as well as patients in a (pre)terminal phase, bad general condition, dementia or confusion severe enough to compromise the testing. The use of inhalation corticosteroids, low dose aspirin (as anti-aggregating medication) and paracetamol was allowed. Patients were included within 72 h after admission.

The study protocol was approved by the IRB and all patients (or proxy) gave written informed consent. The RCT was registered as ISRCTN58517443.

### Intervention and treatment

Patients were randomized, using numbered containers, to receive piroxicam 10 mg or placebo daily for ten days. All patients, except those already using proton pump inhibitors, also received oral ranitidine 150 mg daily to avoid gastro-intestinal side effects. The medical staff was blinded to treatment allocation and patients received standard care. Patients were evaluated until discharge with a maximum of 21 days after treatment allocation.

### Measurements

Primary outcomes were changes in muscle mass, muscle performance, mobility as evaluated by the Elderly Mobility Scale (EMS [8]), and evolution of cyto-/chemokines and Hsp. Explanatory variables included type of infection, comorbidity and general patient parameters such as age, gender, height and weight, as well as basal activities of daily living at admission (bADL; modified scale according to Katz et al.) [9], pre-admission instrumental ADL (iADL; 7-point Lawton scale) [10], and complete biochemical and hematological profile, including renal function (Cockcroft & Gault formula) [11]. Safety outcomes were evolution of renal function, side effects and mortality.

Samples were taken at baseline, after 1, 2 and 3 days, and after 1, 2 and 3 weeks. Peripheral venous blood was collected in the morning after overnight fasting. Clinically relevant parameters were immediately determined. Serum was stored at -20°C for determination of cyto-/chemokines and circulating Hsp. EDTA anticoagulated blood was used for immediate determination of intracellular Hsp in mononuclear cells. For technical reasons intracellular Hsp was not determined on day 3.

#### Muscle performance: Grip strength, fatigue resistance and grip work

Grip strength (GS) and fatigue resistance (FR) were measured within 72 h after admission using a Martin Vigorimeter device (Elmed, Addison, USA) by specially trained physiotherapists as previously described [12]. Briefly, the patients were instructed to squeeze the rubber bulb of the Vigorimeter as hard as possible, with the elbow flexed at 90°. The best result of three trials for each hand was recorded. The patients were then asked to maintain a maximal pressure as long as possible, under repeated verbal stimulations by the physiotherapist, and the time (in seconds) until grip strength decreased to 50% of its maximum value was recorded for each hand. Grip work (GW) was calculated as reported previously [13]: GW = (GSx0.75)xFR. For GS, FR and GW the mean values of both hands were used.

#### Lean body mass

Lean body mass (LBM) was assessed by measuring naturally occurring isotopic ^40 ^K in a whole body counter. ^40 ^K represents 0.012% of total K and is the major source of corporeal radioactivity. The whole body counter used (Nuclear Enterprise, UK) consists of four 10 × 15 cm thallium activated sodium iodine crystals, two above and two beneath an examination bed of perspex. The detectors can be moved along the axis XYZ to achieve the best counting geometry. To keep the background as low as possible, the detectors and examination bed are included in a closed room built with steel from a boat sunken before the first nuclear essays. The background is measured for 15 h the night before the examination. The activity of the patient is measured for 30 min in spectrometric mode; the background is subtracted and the ^40 ^K peak (1.46 MeV) is quantified. Four boxes each containing 500 g of KCl are counted for 30 min in front of each detector. The ^40 ^K activities are summed for the reference samples and for the patient, allowing to calculate the patient's potassium pool.

More than 95% of total body potassium is intracellular, found chiefly in muscle and viscera, and the potassium pool is a good estimator of LBM [14]. LBM was calculated as previously described [15] and expressed in kg.

#### Cytokine determinations

Serum levels of 25 different cyto-/chemokines were measured simultaneously according to the manufacturer's indications (Multiplex Bead Immunoassay, Biosource International, Nijvel, Belgium) including: IL-1β, IL-1RA, IL-2, IL-2R, IL-4, IL-5, IL-6, IL-7, IL-10, IL-12, IL-13, IL-15, IL-17, CCL2/MCP-1, CCL3/MIP-1α, CCL4/MIP-1β, CCL5/RANTES, CCL11/Eotaxin, CXCL8/IL-8, CXCL9/MIG, CXCL10/IP-10, TNFα, IFN-α, IFN-γ and GM-CSF (for full names and sensitivities see legend of Table 1).

**Table 1:** Participants' baseline characteristics

	**Placebo **N = 14	**Piroxicam **N = 14
**General characteristics**		

Age, years	82.5 (79.5-86.5)	85.0 (76.8-91.0)

Gender, number (%) female	10 (71.4%)	9 (64.3%)

BMI, kg/m^2^	23.6 (18.3-26.3)	26.2 (20.1-28.2)

Proportion BMI < 20	4 (28.6%)	2 (14.3%)

Proportion BMI > 25	5 (35.7%)	8 (57.1%)

bADL, 24 point Katz scale	18.0 (10.3-20.3)	12.0 (9.5-19.5)

iADL, 7 point Lawton scale	1.5 (0.3-3.5)	2.0 (1.0-4.0)

EMS, 20 point scale	5.5 (1.0-8.0)	2.5 (0.0-8.5)

**Type of infection & comorbidity**		

- Respiratory, number	8 (57.1%)	6 (42.9%)

- Urinary, number	1 (7.1%)	4 (28.6%)

- Gastro-intestinal, number	3 (21.4%)	1 (7.1%)

Other, number	2 (14.3%)	3 (21.4%)

Number of diagnoses	7.0 (4.8-10.3)	8.0 (5.5-11.0)

**Biochemical parameters**		

CRP, mg/L	89.0 (40.5-156.3)	78.0 (29.8-235.3)

Fibrinogen, mg/dL	651.0 (489.3-752.5)	594.0 (482.5-710.0)

Hb, g/dL	12.7 (11.9-13.8)	12.1 (10.5-13.7)

Prealbumin, g/dL	0.14 (0.09-0.17)	0.14 (0.10-0.18)

GFR, mL/min	54.9 (38.4-65.5)	47.3 (31.6-56.4)

**Skeletal muscle evaluations**		

GS*/body weight (kPa/kg)	0.24 (0.11-0.43)	0.44 (0.24-0.63)

FR*/body weight (sec/kg)	0.21 (0.03-0.54)	0.37 (0.21-0.78)

GW*/body weight (kPa × sec/kg)	3.1 (0.1-8.2)	7.5 (2.7-17.5)

LBM (kg)	24.0 (17.9-30.6)	26.1 (24.9-30.3)

Data represent median values (interquartile range) for continuous variables and numbers (percentage) for categorical variables. No significant difference was observed for any of the parameters.

BMI = body mass index. bADL = basic activities of daily living. iADL = instrumental activities of daily living. CRP = C-reactive protein. Hb = hemoglobin. GFR = glomerular filtration rate calculated with the Cockcroft & Gault formula. GS = grip strength. FR = fatigue resistance. GW = grip work. LBM = lean body mass. *(mean of both hands).

#### Sandwich ELISA for determination of RANTES and of hsp in serum

RANTES levels were above detection limit with the Multiplex Assay and were further analyzed using a commercial ELISA kit (Invitrogen, Camarillo, USA). The standard curve ranged from 0 to 2000 pg/mL (sensitivity of 3 pg/ml).

Hsp27 and Hsp70 in serum were detected using commercial ELISA kits (StressGen, Victoria, Canada). The standard curve ranged from 0 to 12.5 ng/mL (sensitivity of 0.09 ng/ml) for Hsp70, and from 0 to 25 ng/ml (sensitivity 0.39 ng/ml) for Hsp27.

For both ELISA kits all reagents, dilutions and calculations were applied according to the manufacturer's instructions.

#### Flow cytometric determination of intracellular Hsp27 and Hsp70

Hsp27 and Hsp70 were quantified in peripheral blood cells using flow cytometry as previously reported [16]. Briefly, cells were incubated with anti-CD14 antibody (Becton Dickinson, San Jose, Ca) for 15 min, washed in PBS containing 1% BSA, and fixed in reagent A (IMTEC Diagnostics, Antwerpen, Belgium) for 15 min. After washing, the cells were permeabilized with reagent B (IMTEC Diagnostics) and at the same time incubated with the primary Hsp-specific antibody (clone G3.1, SPA-800FI for Hsp27 and clone C92F3A-5, SPA-810 for Hsp70; StressGen, Victoria, Canada). In negative controls, an isotype matched goat IgG (Becton Dickinson, San Jose, Ca) was used as the primary antibody. After 15 min incubation, cells were washed, and 500 μl of facsflow solution Becton Dickinson, San Jose, Ca) were added in the case of Hsp27. For Hsp70 the labeled cells were further re-suspended in reagent B and the secondary antibody (Goat anti-mouse gG, IMTEC Diagnostics), and incubated for 15 min. Cells were washed and 500 μl of Facsflow solution were added. The samples were analyzed immediately or within a few hours (stored at 4°C) using the flow cytometer Epics-XL-MLC.

#### Heat shock procedures

Intracellular Hsp levels were determined both with and without heat challenge (HC and WHC respectively), at room temperature, as previously described [17]. Briefly, for HC, Petri dishes containing the cells were heated at 42°C for 1 h. After HC, adherent cells were allowed to recover for 16 h at 37°C in a 5% CO_2 _incubator. Cells were then detached, washed, counted, and their viability assessed. At least 85% of the cells survived after exposure to 42°C for 1 h.

### Statistical analysis

Analyses were done using IBM SPSS statistics 19.0 (SPSS Inc, Illinois, USA). Data are reported as median ± interquartile range. As several datasets for muscle performance and cytokines were not normally distributed non-parametric procedures (with exact testing) were used: Spearman's rho for correlations, Mann Whitney *U *test for unpaired comparisons, Friedman test for overall time effects, and Wilcoxon's signed rank test for changes relative to baseline. Differences in the patterns of relationships between changes in cytokines during hospitalization were analyzed using the McNemar test. Differences in proportions were analyzed using the Pearson Chi-square test. Significance was set a priori at two-sided *p *< 0.05.

## Results

### General characteristics

**Figure 1:**
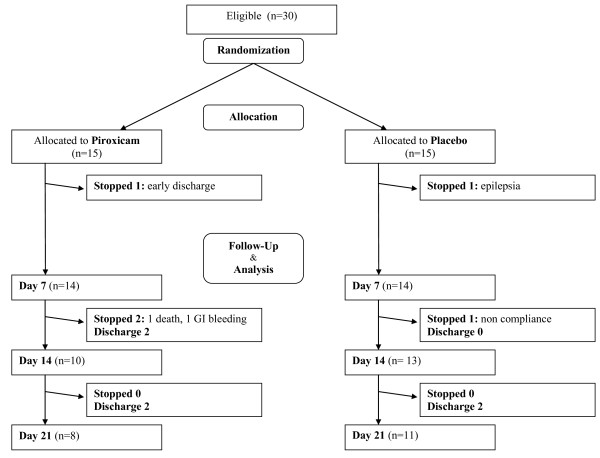
**Flow chart**. Numbers of patients at randomization, treatment allocation and each time point during follow-up.

30 Caucasian patients, median age 84.5 years (range 70-94), were included (see Figure 1). One patient in each treatment arm had to be withdrawn rapidly after randomization, and no follow-up evaluation of the primary outcome measures was possible (one patient with rapidly improving respiratory infection in the piroxicam group was discharged from the hospital on his demand; one patient with epileptic seizures in the placebo group was withdrawn from the study). As shown in Table 1 and 2, no significant differences were observed at baseline between the two treatment groups. Patients were predominantly female, highly dependent in bADL and iADL, a majority (21 patients) was malnourished (based on serum prealbumin < 0.20 g/dL), had a moderately reduced renal function and showed multiple comorbidity.

**Table 2:** Baseline levels of cytokines and heat shock proteins

	sensitivity	**Placebo **N = 14	**Piroxicam **N = 14
**Cytokines **in pg/mL			

IL-1beta	< 15	31.0 (21.0-112.5)	14.9 (14.9-65.3)

IL-1RA	< 20	245.0 (134.5-537.0)	412.0 (177.5-615.0)

IL-2	< 15	14.9 (14.9-35.0)	14.9 (14.9-14.9)

IL-2R	< 40	248.0 (150.5-346.5)	427.5 (207.5-595.0)

IL-4	< 5	4.9 (4.9-22.5)	4.9 (4.9-10.5)

IL-5	< 5	LO	LO

IL-6	< 5	22.0 (14.5-89.5)	19.0 (6.5-160.5)

IL-7	< 25	24.9 (24.9-32.5)	24.9 (24.9-40.5)

IL-10	< 3	5.0 (3.0-29.0)	5.0 (3.0-6.0)

IL-12	< 6	169.0 (107.0-253.0)	160.0 (115.0-274.3)

IL-13	< 6	LO	LO

IL-15	< 25	39.0 (24.9-134.5)	25.0 (24.9-60.0)

IL-17	< 20	LO	LO

GM-CSF	< 5	4.9 (4.9-44.0)	4.9 (4.9-8.0)

TNF-alpha	< 5	4.9 (4.9-9.5)	4.9 (4.9-8.5)

IFN-alpha	< 25	38.0 (25.5-66.5)	41.5 (24.9-55.3)

IFN-gamma	< 2	LO	LO

MCP-1/CCL2	< 8	778.0 (561.0-1228.5)	818.0 (458.5-1017.0)

MIP-1alpha/CCL3	< 15	44.0 (21.5-96.5)	49.0 (30.0-66.5)

MIP-1beta/CCL4	< 10	123.0 (62.0-186.0)	106.0 (56.0-192.0)

RANTES/CCL5	< 20	HI	HI

Eotaxin/CCL11	< 5	76.0 (64.5-155.5)	79.0 (63.8-127.5)

IL-8/CXCL8	< 3	132.0 (60.0-294.5)	63.0 (53.5-276.0)

MIG/CXCL9	< 20	76.0 (61.0-186.0)	133.0 (82.0-180.5)

IP-10/CXCL10	< 5	44.0 (32.0-65.0)	53.0 (46.0-106.0)

**Serum Hsp**, ng/mL			

- Hsp27	< 0.39	48.9 (14.3-72.3)	20.1 (15.8-53.5)

- Hsp70	< 0.09	1.1 (0.6-1.7)	1.5 (0.9-1.7)

**Intracellular Hsp**, MFI (flow cytometry)			

- Hsp27			

Lymphocytes - WHC		2.4 (1.6-4.3)	2.4 (1.5-4.9)

- 42°C (HC)		5.2 (3.4-7.6)	5.0 (4.1-6.5)

Monocytes - WHC		28.1 (25.3-42.9)	29.0 (16.0-43.1)

- 42°C (HC)		40.3 (29.2-51.2)	41.2 (29.8-59.9)

- Hsp70			

Lymphocytes - WHC		2.1 (1.6-4.4)	2.5 (1.5-4.3)

- 42°C (HC)		11.9 (7.8-14.9)	13.5 (5.1-18.0)

Monocytes - WHC		13.5 (8.2-26.8)	12.7 (8.1-18.8)

- 42°C (HC)		244.1 (149.9-332.1)	211.0 (160.5-343.4)

Data represent median values (interquartile range). *P*-values refer to unpaired comparisons by Mann Whitney *U *test. No significant difference was observed for any of the parameters. GM-CSF = granulocyte macrophage colony-stimulating factor; IL = interleukin. IL-1RA = IL-1 receptor antagonist. TNF-alpha = tumor necrosis factor alpha. IFN = interferon. IL-2R = IL- 2 receptor. For chemokines old and new names are indicated, separated by a forward slash; MCP = monocyte chemoattractant protein. MIP = macrophage inflammatory protein. RANTES = Regulated upon Activation, Normal T-cell Expressed, and Secreted. MIG = monokine induced by interferon gamma. IP-10 = interferon γ-inducible protein 10. Hsp = heat shock protein(s), MFI = Mean Fluorescence Intensity, WHC = without heat challenge, 42°C(HC) = heat challenge, LO = below lower detection limit, HI = above upper detection limit.

No significant gender difference was observed for GS, FR or GW. GS tended to be lower in women (*p *= 0.068). After correcting GS for body weight, this tendency disappeared. Data from men and women were pooled and all analyses were done for GS, FR and GW per kg body weight. Women had higher baseline levels of CRP than men (*p *= 0.022), but the proportion of women was the same in both groups and no differences in serum CRP levels were observed between treatment groups for women and men respectively. CRP decreased significantly in both treatment groups (*p *< 0.001 for placebo and for piroxicam at weeks 1, 2 and 3). LBM was measured in 24 of the 28 follow-up patients. Four patients, two in each treatment arm, could not be evaluated having undergone other nuclear medicine examinations. Throughout the study, IL-5, IL-13, IL-17 and IFNγ levels remained below the detection limit in more than 70% of the patients in both groups. Further analyses thus concerned the remaining 21 cyto-/chemokines.

### Changes in mobility and muscle performance over time

**Figure 2:**
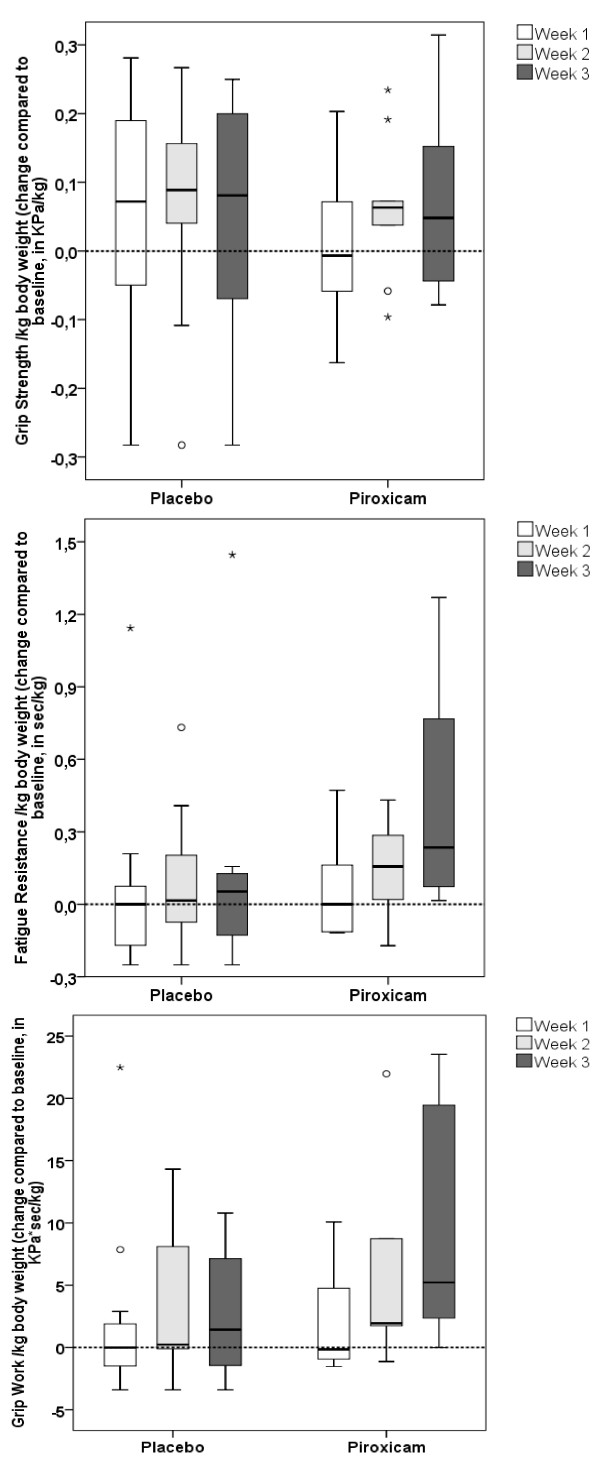
**a-b-c: Changes in GS, FR and GW at weeks 1, 2 and 3 with placebo and piroxicam respectively**.

During follow-up EMS scores improved significantly after two weeks with piroxicam (*p *= 0.021), with a positive tendency maintained at week 3 (*p *= 0.063), whereas a positive tendency was observed in the placebo group at week 2 and no effect at week 3 (*p *= 0.054 and *p *= 0.565 respectively). LBM and GS did not change significantly in either group. FR increased in the piroxicam group. A significant overall time effect was observed (*p *= 0.004, Friedman test over 3 week period), with a tendency for improvement compared to baseline at week 2 and a significant change at week 3 (*p *= 0.059 and *p *= 0.018 respectively, Wilcoxon's signed rank test). GW also improved with piroxicam. A significant overall time effect was observed (*p *= 0.011, Friedman test over 3 week period), with improvements at weeks 2 and 3 compared to baseline (*p *= 0.013 and *p *= 0.028 respectively, Wilcoxon's signed rank test) (Figure 2). Body weight and LBM were correlated at baseline (r = 0.902, *p *< 0.001, Spearman's rho). Consequently, when correcting GS, FR and GW for LBM instead of body weight, comparable results with the same level of significance were observed (for GS: no significant change; for FR: *p *= 0.004 for overall time effect, and compared to baseline *p *= 0.074 and *p *= 0.018 at weeks 2 and 3 respectively; for GW: *p *= 0.011 for overall time effect, and compared to baseline *p *= 0.013 and *p *= 0.028 at week 2 and 3 respectively). FR and GW showed no change in the placebo group. However, the changes in FR or GW showed no significant difference between groups.

### Relationships between baseline inflammation and changes in muscle performance

**Figure 3:**
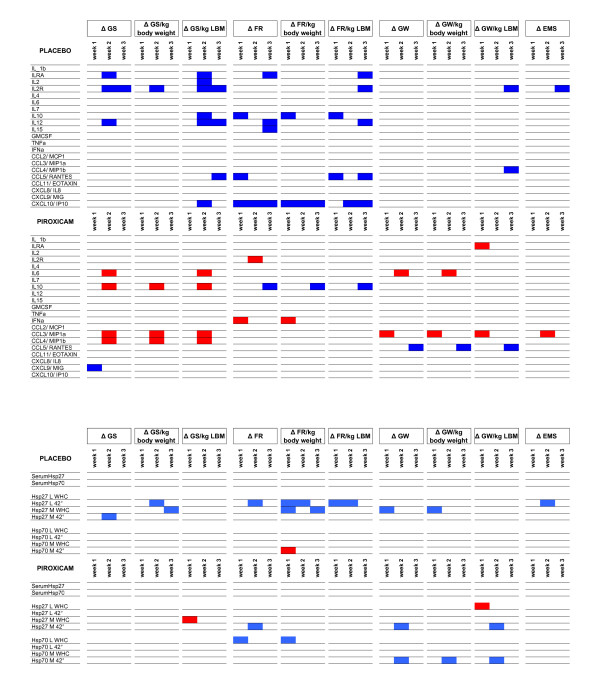
**Spearman's rho correlations between baseline serum levels of cytokines (Figure 3a) or baseline levels of extra-and intra-cellular Hsp (Figure 3b) and subsequent changes in muscle parameters (raw data as well as results corrected by kg body weight and by kg lean body mass)**. Significant correlations with *p *< 0.05 (two-sided) are indicated in red when positive, in blue when negative.

Correlations between baseline levels of cytokines and subsequent changes in muscle parameters were exclusively negative in the placebo group and were positive in the piroxicam group, except for IL-10, RANTES and MIG. The cytokines that were correlated with changes in muscle parameters differed largely between the treatments groups (Figure 3a). Baseline Hsp27 WHC and after HC showed multiple, exclusively negative correlations with the evolution of GS, FR, GW and EMS in the placebo group. In the piroxicam group Hsp27 WHC showed positive correlations, and Hsp27 after HC negative correlations. Hsp70 WHC and after HC showed several negative correlations in the piroxicam group and a positive correlation in the placebo group, realizing a rather inversed pattern of relationships (Figure 3b).

### Relationships between changes in inflammatory markers and changes in muscle performance

**Figure 4:**
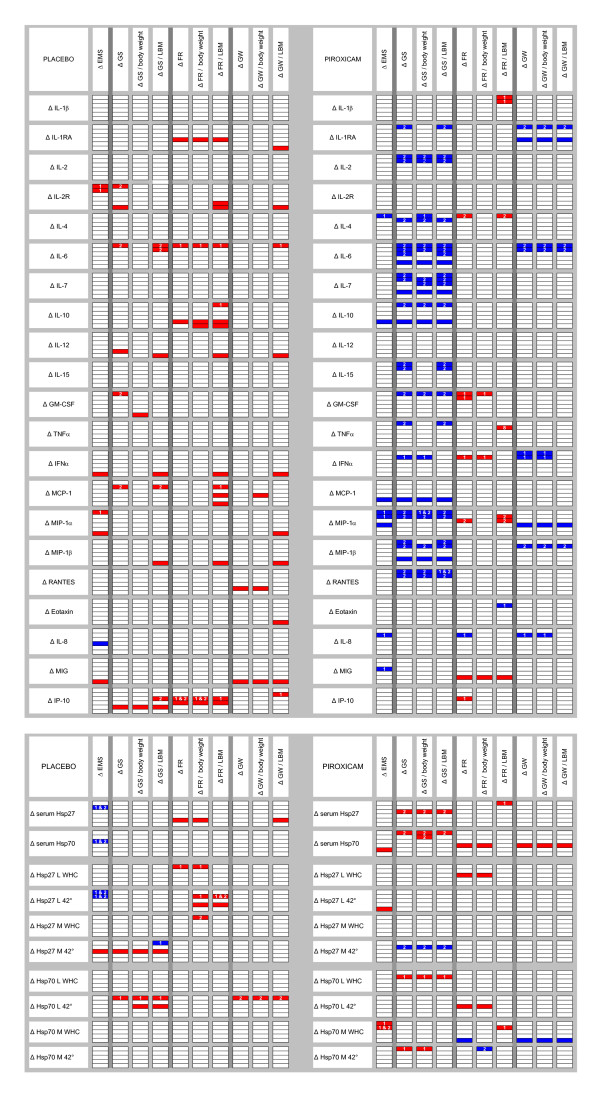
**Spearman's rho correlations between changes in cytokines (Figure 4a) or changes in extra-and intra-cellular Hsp (Figure 4b) and changes in muscle parameters (raw data as well as results corrected by kg body weight and by kg lean body mass)**. Significant correlations with *p *< 0.05 (two-sided) are indicated in red when positive, in blue when negative. Serum levels of cytokines and Hsp were measured at 6 follow-up time-points (changes to baseline indicated by 6 rectangles for each cytokine in each column). Intracellular Hsp were measured at 5 follow-up time-points (changes to baseline indicated by 5 rectangles for each). Muscle parameters were evaluated at 3 follow-up time-points. Correlations were computed between early changes (first days) in cytokines or Hsp and changes in muscle parameters at week 1 and 2. When correlations with these early changes were significant, white numbers in the corresponding rectangle indicate the corresponding time-point of the concerned muscle parameter. The lower three rectangles indicate the concomitant changes compared to baseline at weeks 1, 2 and 3 for both parameters concerned.

When correlations were computed between changes compared to baseline (Figure 4a), an overall difference in the pattern of relationships was observed in the piroxicam group compared to placebo. Whereas various positive correlations between the changes of inflammatory and muscle parameters occurred in the placebo group, these correlations were negative in the piroxicam group for GS and GW, and, although the relationships for FR remained positive, they concerned other inflammatory parameters. The correlations in the piroxicam group were mainly found for early observations.

With respect to Hsp, serum Hsp70 decreased (*p *= 0.024), whereas Hsp27 WHC in monocytes increased (*p *= 0.016) in the piroxicam group. The changes compared to baseline were significantly different between groups for Hsp27 in monocytes (*p *= 0.021), but not for serum Hsp70. Hsp70 WHC in monocytes decreased in both groups (*p *= 0.047 for placebo and *p *= 0.028 for piroxicam). Changes in Hsp were heterogeneously related to the changes in muscle parameters (Figure 4b).

### Changes in general parameters

Serum potassium significantly increased in both groups over time and serum levels were comparable at each time point. Prealbumin increased in both treatment groups after 1 week (*p *= 0.025 piroxicam and *p *= 0.034 placebo).

GFR did not change over the whole study period. Nevertheless, a temporary decrease was observed at week 1 compared to baseline in the piroxicam group (*p *= 0.022) with a tendency for lower GFR compared to placebo (*p *= 0.071). There was a tendency for a higher incidence in side effects in the piroxicam group (*p *= 0.075), but no difference in overall clinical evolution, length of stay or mortality (Table 3).

**Table 3:** Clinical evolution

		Placebo	Piroxicam	p
**Clinical evolution **(number of patients)				

	Return to premorbid clinical status	6	10	0.127*
	
	Persistent decrease in clinical status	8	4	

Side effects (attributable to NSAID)				

	None	13	8	0.075*

	Possible	1	3	

	Probable	0	3	

**Length of stay **(days; median, range)		24.5 (19-64)	29.0 (9-75)	0.836^†^

**Death**		1	2	0.541*

*Pearson Chi square test. ^†^Mann Whitney *U *test.

## Discussion

In this cohort of frail geriatric patients hospitalized with acute infection-induced inflammation we observed a better recovery of mobility, assessed by EMS, that paralleled a better recovery of muscle performance, evaluated by fatigue resistance and grip work, in the patients randomized to double blind controlled treatment with piroxicam. These improvements were not related to LBM that did not change significantly during the study period, as evaluated by total body potassium. We have previously shown that muscle function is impaired in geriatric hospitalized patients with inflammation and that the decreased muscle function was not explained by muscle mass estimations using anthropometric measures [3]. We further demonstrated that inflammatory patients did not recover muscle strength and fatigue resistance during hospitalization despite adequate treatment of the underlying condition and a significant decrease in inflammatory markers. These observations hold true in the present study where no significant improvement in muscle function of inflammatory patients was observed in the placebo group despite a significant decrease in CRP serum levels, and where the increases in FR, GW and mobility observed with piroxicam were not related to whole body muscle mass as evaluated by total body potassium pool.

Muscle performance is difficult to evaluate in hospitalized geriatric patients. We previously developed a FR test easy to use in frail geriatric patients in order to evaluate muscle endurance [12]. This test has been able to detect differences between inflammatory and non inflammatory patients in the absence of changes in the classical GS test that evaluates a brief maximal contraction [3]. GW that integrates GS and FR probably is the most clinically relevant measure, as it indicates the capacity of sustaining strength in time, required by many activities of daily living [4]. Correction for body weight takes into account that physical efforts are determined not only by the action or object itself, but also by a person's own body weight. Furthermore, it is important that these measures translate into functional improvements. Here, we demonstrate significant increases in FR and GW corrected for body weight and EMS scores with piroxicam, but not with placebo. The EMS measures basic mobility reflecting the capacity of our acutely ill patients to get out of bed and move around. Not only does this indicate better performance, but it also reflects a decreased risk for the vicious cycle of acute weakness and fatigue leading to immobility, disuse atrophy, further weakness and functional decline.

Loss of muscle performance in our patients occurred in the absence of significant loss of muscle mass. Skeletal muscle contractility can be influenced by cytokines directly, independent of changes in muscle mass and protein content. Monocyte inflammatory products released during sepsis, especially TNFα, have been shown to impair skeletal muscle contractility through an increase in mitochondrial or cytosolic production of reactive oxygen species (ROS) [18], and calcium-activated calpains lead to Z disk disruption and release of myofilaments, a phenomenon that has been shown to be up regulated in sepsis [18]. In addition, cytokine-induced sickness behaviour and fatigue, mediated by the central nervous system, probably contribute to lower muscle endurance. Here, we investigated the role of a large panel of cyto-/chemokines. Higher baseline levels of several cyto-/chemokines were correlated to worse evolution of muscle parameters in the placebo group, and to better evolution in muscle function in the piroxicam group, suggesting a more pronounced effect of NSAID treatment when baseline inflammation is more severe.

In a previous semi-blinded trial we showed that FR improves significantly (> 60%) following treatment with a selective COX-2 inhibitor [5], but cytokines were not followed up during the first week. Here, we show that changes in muscle parameters at week 1 and week 2 are mainly correlated to early changes in cytokine levels during the first days. Exclusively positive correlations in the placebo group reflect decreases in cytokine levels that parallel decreases in muscle performance despite adequate treatment of the infectious disease. With piroxicam, negative correlations indicate that decreases in cytokine levels are related to improved muscle performance and it is noteworthy that early decreases in IL-6 serum levels with piroxicam were correlated with increased GW performance at week 2. The persistence of positive correlations between changes in cytokines and changes in FR are unexpected, but concern other cyto-/chemokines than in the placebo group and may reflect immunomodulatory rather than simply cytokine-inhibitory effects of piroxicam. COX-specific effects of piroxicam [19] and immunomodulatory and COX-independent effects have been described and discussed elsewhere, in general [20,21], and in this patient cohort in particular [22].

NSAID treatment may partially induce the human heat shock response [21]. In our patients basal expression of Hsp27 in monocytes WHC, but stimulated by infection-induced inflammation, was positively correlated with FR and LBM at baseline and significantly increased by treatment with piroxicam compared to placebo. Activation of stress genes by NSAID, including piroxicam, has previously been described [6]. Hsp27 expression is abundant not only in PBMC, but also in skeletal muscle; it has been shown to increase with aging in muscle homogenate samples at rest [23] and has been linked to improved cardiac muscle function after ischemic injury [24]. Hsp27 thus might play a direct role in muscle protection in our patients if its expression in PBMC is a good substitute for its expression in myocytes. More invasive biopsy studies are required to elucidate this hypothesis.

It cannot be excluded that some contrasts might have been missed due to dropouts (thus increasing the risk for a type-2 error in our study). The number of patients recruited here was based on a sample size calculation using data of a previous study demonstrating significant effects of celecoxib on muscle endurance and mobility within and between groups with the same sample size (n = 15 per group) [5]. Even though the mean length of stay was 27 days in the geriatric ward where the present study was carried out, our cohort suffered from losses due to almost twice as many discharges before the scheduled follow up at 3 weeks in the piroxicam group (46%) compared to the placebo group (25%). The time of discharge was decided independently of the study (by independent physicians), and it might be possible that patients with an early discharge presented with better recovery of muscle performance and that significant between-group effects may have been missed due to insufficient sample size. However, the study design did neither include prolonged hospitalization nor follow up evaluations after discharge. The latter may be a relevant attention point for future study designs. Nevertheless, the present sample size was sufficient to detect significant within group improvements. *Post hoc *power calculations indicated power > 80% for detecting the observed changes (with alpha = 0.05) in EMS, FR and GW in 15 patients over 3 weeks. For GS however, power was lower (40%).

## Conclusion

The present double blind RCT suggests a better initiation of recovery in muscle endurance (FR and GW corrected by body weight) and mobility with the COX non selective NSAID piroxicam in geriatric patients hospitalized with acute infection, and confirms our earlier findings in a single blinded study with the COX-2 selective celecoxib. Anti-inflammatory strategies in these patients deserve more attention in clinical decision making, since grip work capacity per kg body weight and mobility represent clinically relevant outcomes with respect to performing daily activities. Modulation of the cytokine network and increases in cellular expression of Hsp27 appear to participate in the underlying mechanisms that are favorably influenced by treatment. The safety profile of piroxicam appears acceptable when associated with protection of the gastric mucosa.

## Competing interests

Beyer I, Mets T have been investigators in clinical trials sponsored by Pfizer and have received travel fees from Pfizer and other pharmaceutical companies. The present study was an investigator initiated trial receiving no sponsoring and the study medication was produced by the hospital's pharmacy.

## Authors' contributions

Mets T., Bautmans I., Njemini R, and Beyer I. wrote the study protocol. Bautmans I. trained the study physiotherapists. Beyer I. recruited the patients, applied the study protocol, prescribed the study medication, assured transport of the samples, analyzed the data and wrote the paper. Bautmans I. and Mets T. critically appraised the analyses and manuscript. Njemini R. measured cytokines and Hsp under supervision by Demanet C. Bergmann P. evaluated LBM using ^40^K measurement. Mets T. coordinated the study. All authors read and approved the final manuscript.

## Pre-publication history

The pre-publication history for this paper can be accessed here:

http://www.biomedcentral.com/1471-2474/12/292/prepub
